# Two Cases of Lithotomy

**Published:** 1882-01-20

**Authors:** Alexander F. Durham

**Affiliations:** Georgia


### Southern Medical Record:

EDITORS:

T. S. POWELL, M.D. W. T. GOLDSMITH, M.D. R. C. WORD, M.D.

*R. C. WORD, M.D., Managing Editor.*

☞All Communications and Letters on Business connected with the RECORD must be addressed to the Managing Editor.

VOL. XII. ATLANTA, GA., JANUARY 20, 1882. No. 1.

### ORIGINAL AND SELECTED ARTICLES.

### TWO CASES OF LITHOTOMY.

BY ALEXANDER F. DURHAM, M. D., OF GEORGIA.

CASE I.—Miles, a dark ginger-cake colored boy, aged nine years, was brought to me for treatment in the summer of 1875. All the symptoms of vesicular calculus were present in his case. From his father I obtained the following history: From his earliest infancy he was unable to retain his urine, and when old enough was continually "tugging and working" with his penis, passing water incessantly, and occasionally blood. When brought to me he presented the following symptoms: Was exceedingly emaciated, almost a living skeleton, weighing not over 35 pounds; rough, ash-colored cheeks; tongue rather dry; thirst and anorexia. The penis, especially the glands, were abnormally developed. The slightest flow of urine was accompanied by distressing pains and uneasiness. The metallic sound introduced met with firm resistance at the neck of the bladder, evidently due to the presence of a stone. On making a thorough examination the calculus seemed to fill the entire cavity of that organ. Long existence of inflammation had caused thickening, induration and contraction of its coats.

I put him on a constitutional treatment for a short time, to brace up the system. When this was done, I proceeded, with the aid of a few intelligent assistants, (there being no physician present but myself,) to remove the stone by the bilateral operation. Unfortunately the stone was so large that I had to crush it before extracting it. It was impossible to extend the operation without greatly increasing the danger of the patient's life.

He recovered in fifteen or twenty days without an untoward symptom, and when I last heard from him there was no indication whatever of a return of gravel.

CASE II.—Daughter of Bell McCommon, black, aged six years. This child was brought to Dr. W. M. Durham and myself in April last. The symptoms and history much the same as in the first case, viz: Emaciation (not so extreme), stillicidium, incessant pain in region of the bladder; urine occasionally tinged with blood, otherwise normal. Pudenda excessively developed, and almost constantly manipulated by the patient.

A stone of unusual size was readily detected by the introduction of a metallic sound. The patient was kept on constitutional treatment for a short time.

On the 12th of the following May, assisted by Drs. W. M. and John L. Durham, I proceeded to remove the stone by the subpubal operation. A slightly curved bistoury guided by a grooved director was introduced into the bladder, then withdrawing it a slit was made from behind forwards the whole length of the upper portion of the urethra. The calculus proving to be too large to be passed through the first incision two others were made, one on each side of the first, and after a good deal of difficulty the extraction of the stone was accomplished. It measured exactly one and five-eighths ($1\frac{5}{8}$) of an inch in length, and five-eighths ($\frac{5}{8}$) of an inch in its short diameter, and weighed precisely 260 grains.

The patient made a good recovery, but it was some two or three months before complete control over the sphincter of the bladder was regained.

My opinion is that both were cases of conginetal calculus.

